# Effects of Vascular Risk Factors on the Association of Blood-Based Biomarkers with Alzheimer’s Disease

**DOI:** 10.18103/mra.v11i9.4468

**Published:** 2023-10-04

**Authors:** SS Hoost, AM Brickman, JJ Manly, LS Honig, Y Gu, D Sanchez, D Reyes-Dumeyer, RA Lantigua, MS Kang, JL Dage, R Mayeux

**Affiliations:** 1. Taub Institute for Research on Alzheimer’s Disease and the Aging Brain, Vagelos College of Physicians and Surgeons, Columbia University, New York, NY; 2. G.H. Sergievsky Center, Vagelos College of Physicians and Surgeons, Columbia University, New York, NY; 3. Department of Neurology, Vagelos College of Physicians and Surgeons, Columbia University, and the New York Presbyterian Hospital, New York, NY; 4. Department of Medicine, Vagelos College of Physicians and Surgeons, Columbia University, and the New York Presbyterian Hospital, New York, NY; 5. Stark Neurosciences Research Institute, Indiana University School of Medicine, Indianapolis, IN; 6. Department of Neurology, Indiana University School of Medicine, Indianapolis, IN; 7. Indiana Alzheimer’s Disease Research Center, Indiana University School of Medicine Indianapolis IN

## Abstract

**Background:**

Comorbidities may influence the levels of blood-based biomarkers for Alzheimer’s disease (AD). We investigated whether differences in risk factors or comorbid conditions might explain the discordance between clinical diagnosis and biomarker classifications in a multi-ethnic cohort of elderly individuals.

**Aims:**

To evaluate the relationship of medical conditions and other characteristics, including body mass index (BMI), vascular risk factors, and head injury, with cognitive impairment and blood-based biomarkers of AD, phosphorylated tau (P-tau 181, P-tau 217), in a multi-ethnic cohort.

**Methods:**

Three-hundred individuals, aged 65 and older, were selected from a prospective community-based cohort for equal representation among three racial/ethnic groups: non-Hispanic White, Hispanic/Latino and African American/Black. Participants were classified into four groups based on absence (Asym) or presence (Sym) of cognitive impairment and low (NEG) or high (POS) P-tau 217 or P-tau 181 levels, determined previously in the same cohort: (Asym/NEG, Asym/POS, Sym/NEG, Sym/POS). We examined differences in individual characteristics across the four groups. We performed post-hoc analysis examining the differences across biomarker and cognitive status.

**Results:**

P-tau 217 or P-tau 181 positive individuals had lower BMI than P-tau negative participants, regardless of symptom status. Symptomatic and asymptomatic participants did not differ in terms of BMI. BMI was not a mediator of the effect of P-tau 217 or P-tau 181 on dementia. Frequencies of other risk factors did not differ between the four groups of individuals.

**Conclusions:**

Participants with higher levels of P-tau 217 or P-tau 181 consistent with AD had lower BMI regardless of whether the individual was symptomatic. These findings suggest that weight loss may change with AD biomarker levels before onset of cognitive decline. They do not support BMI as a confounding variable. Further longitudinal studies could explore the relationship of risk factors with clinical diagnoses and biomarkers.

## Introduction

In individuals with mild cognitive impairment (MCI) or dementia, evidence of cerebral amyloid and tau accumulation accompanying neurodegeneration is necessary to confirm the diagnosis of Alzheimer’s disease (AD)^1^. Blood-based biomarkers, in comparison with established cerebrospinal fluid (CSF) and positron emission tomography (PET) biomarkers, have potential as a less invasive way to detect AD. Blood-based phospho-tau 217 (P-tau 217) and phospho-tau 181 (P-tau 181) are sensitive and specific markers of AD pathology^2–4^, outperforming plasma amyloid-beta (Ab42/40), neurofilament light chain (NfL) and total tau^2,3^. Plasma P-tau 181 and P-tau 217 correlate with CSF P-tau 181 and P-tau 217 and detect AD with similar accuracy^3,4^. Use of plasma biomarkers would make diagnostic information more accessible both in large cohort studies and in clinical settings.

Implementing blood-based biomarkers in the clinic, it is important to be aware of medical conditions or other factors that influence biomarker concentrations and accuracy^5^. Community-based studies tend to have a higher frequency of concomitant medical conditions than clinic-based studies^6^. However, blood-based biomarkers have mostly been studied in clinical trials and clinic-based samples^6^. In addition, it is important to study the performance of biomarkers in ethnically diverse cohorts, which include underrepresented minority groups^5^.

We have previously evaluated 300 community-based individuals chosen for equal representation among ethnic groups divided into four groups based whether positive (POS) or negative (NEG) for plasma P-tau 217 and P-tau 181 concentrations that discriminated AD pathology^2^, and whether symptomatic (Sym) or asymptomatic (Asym) for mild cognitive impairment (MCI) or dementia. The four groups differed by age, *APOE-e4* status, plasma total tau and NfL^7^. The authors found that large discordant groups existed, which were P-tau negative but had cognitive impairment, or P-tau positive but without impairment.

To investigate the discordance between clinical diagnosis and blood-based biomarkers in this cohort, we chose to evaluate whether the four groups varied in terms of risk factors related to dementia, including body mass index (BMI), diabetes mellitus, vascular risk factors and head injury. Change in BMI has been debated as either a risk factor or an effect of AD^8^. A negative association between P-tau and BMI was previously identified in this group^2^. Researchers have identified associations of vascular risk with impaired cognition and dementia both independently from and synergistically with AD biomarkers^9–11^. In a systematic review, traumatic brain injury was associated with increased odds ratio of dementia but not AD^12^. We hypothesized that those with risk factors may be more likely to be symptomatic despite having below threshold levels of AD biomarkers. We performed post-hoc analyses to determine the relationship of risk factors with cognition and with P-tau.

## Methods

The study was approved by the Columbia University Irving Medical Center Institutional Review Board. All participants gave written informed consent. Eligible participants were individuals aged 65 and older who resided in northern Manhattan and were part of the Washington Heights Inwood-Columbia Aging Project (WHICAP) a multiethnic, community-based prospective cohort study. All 300 participants were selected, as previously described^7^, for equal representation from race/ethnicity groups (non-Hispanic White, Hispanic, and non-Hispanic Black) with similar numbers with an AD clinical diagnosis at their last assessment. Race/ethnicity were reported and coded using standardized criteria^13^.

The study design was described in detail elsewhere^14^. Briefly, participants were seen initially for a baseline visit and had follow-up visits every 18–24 months. At each visit, participants received a medical interview, brief neurological exam, and neuropsychological battery, and gave a blood sample. The clinical diagnosis of MCI, AD, other dementia, or no dementia was made at a consensus conference attended by neurologists and neuropsychologists. At follow-up visits, evaluators were blinded to the patient’s previous diagnosis. A clinical diagnosis was assigned using the criteria of the National Institute of Neurological Disorders and Stroke and the Alzheimer’s Disease and Related Disorders Association^15^ and a Clinical Dementia Rating of 0.5 or higher^16^.

Coding of vascular risk factors has been described in previous reports^17,18^. The reliability and validity of self-reported hypertension, diabetes and heart disease has been confirmed in our cohort^19^. Head injury was defined by self-report with loss of consciousness of greater than 30 minutes. Heart disease was defined as a self-reported history of myocardial infarction, congestive heart failure, or another heart condition. Peripheral vascular disease was defined by self-report. BMI (weight in kilograms divided by height in meters squared) was calculated using height and weight measured in person. We used continuous hemoglobin (Hb) A1C as a measure of diabetes mellitus to account for medical management. Education was recorded as total years (0 – 20) attending a school based on self-report.

### Biomarkers.

These methods were previously described^2^. Briefly, centrifuged plasma aliquoted in polypropylene tubes and stored at −80°C was used to measure Aβ42, Aβ40, and total-tau using SIMOA technology (Quanterix, Lexington, MA, USA). The multiplex Neuro 3-plex A kit (#101995), and NfL kit (#103400) were used on 96-well plates. We considered the ratio of Aβ42 to Aβ40 (Aβ42/Aβ40) as the primary amyloid biomarker. The P-tau assays were optimized to measure disease-related differences through the selection of monoclonal antibodies. The P-tau181 assays were performed on a streptavidin small spot plate using the Meso Scale Discovery (MSD) platform. For the P-tau181 assay, Biotinylated-AT270 was used as a capture antibody (anti-P-tau181 antibody) and SULFO-TAG-Ru-4G10-E2 (anti-tau monoclonal antibody) for the detector. The assay was calibrated using a synthetic P-tau181 peptide. For the P-tau217 assays, Biotinylated-IBA493 was used as a capture antibody (anti-P-tau217 antibody) and SULFO-TAG-Ru-4G10-E2 (anti-tau monoclonal antibody) for the detector. The assay was calibrated using a synthetic P-tau217 peptide.

Participants were classified into four groups as previously described^7^, based on P-tau 217 or 181 concentration, positive (POS) or negative (NEG), and based on clinical status, whether symptomatic with MCI or dementia (Sym) or asymptomatic control (Asym). We used ANOVA (for continuous measures) and Chi Square (for categorical measures) to evaluate differences in risk factors across the four groups. We used the False Discovery Rate to account for multiple comparisons. Then, we performed post-hoc analyses to determine the relationship of significant risk factors with P-tau217, P-tau181 and clinical status, including linear and logistic regressions, with and without interaction terms, and regression-based mediation analysis.

## Results

Women represented 67% of the cohort (Table 1). Forty-two percent of participants had cognitive impairment (Table 1). Hypertension and heart disease were the most prevalent comorbidities, at 58% and 44%, respectively (Table 1).

**Figure 1.**
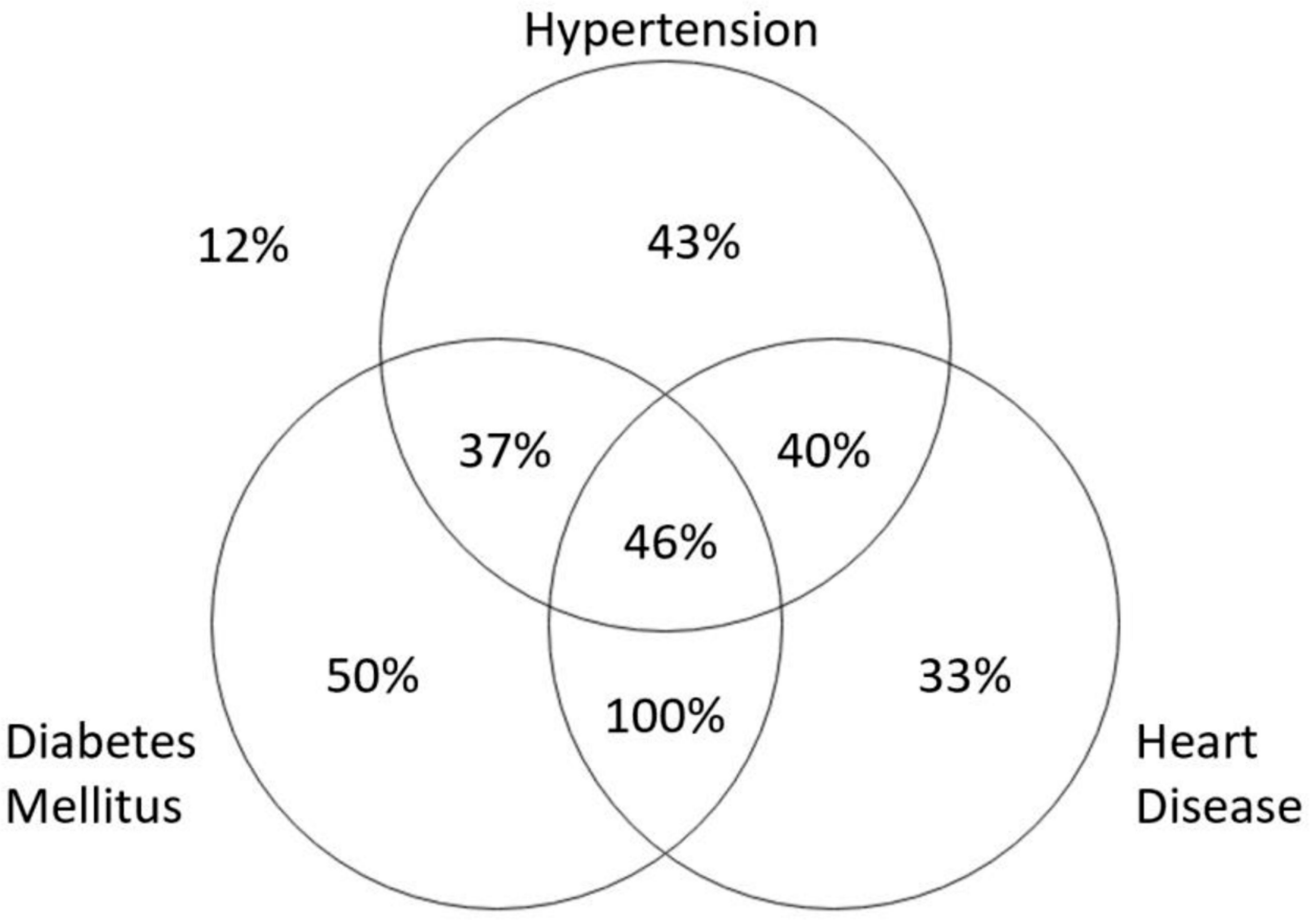
Venn diagram with percentage of participants with each comorbid condition who were symptomatic.

As shown in figure 1, 46% of those individuals with hypertension, diabetes, and heart disease, 50% of those with diabetes alone and 100% of those with diabetes and heart disease were clinically symptomatic regardless of biomarker status. This strongly implicates diabetes as a major contributor to cognitive impairment.

Comparisons of age and sex across the four groups determined by P-tau 217 and P-tau 181 were previously reported^7^. Hb A1C and frequencies of hypertension, heart disease, peripheral vascular disease and head injury did not differ across the four groups determined by P-tau 217 (Table 1) or P-tau 181 (Table 2). BMI differed across the four groups determined by both biomarkers (Table 1, 2).

### Post-hoc analyses

**Figure 2.**
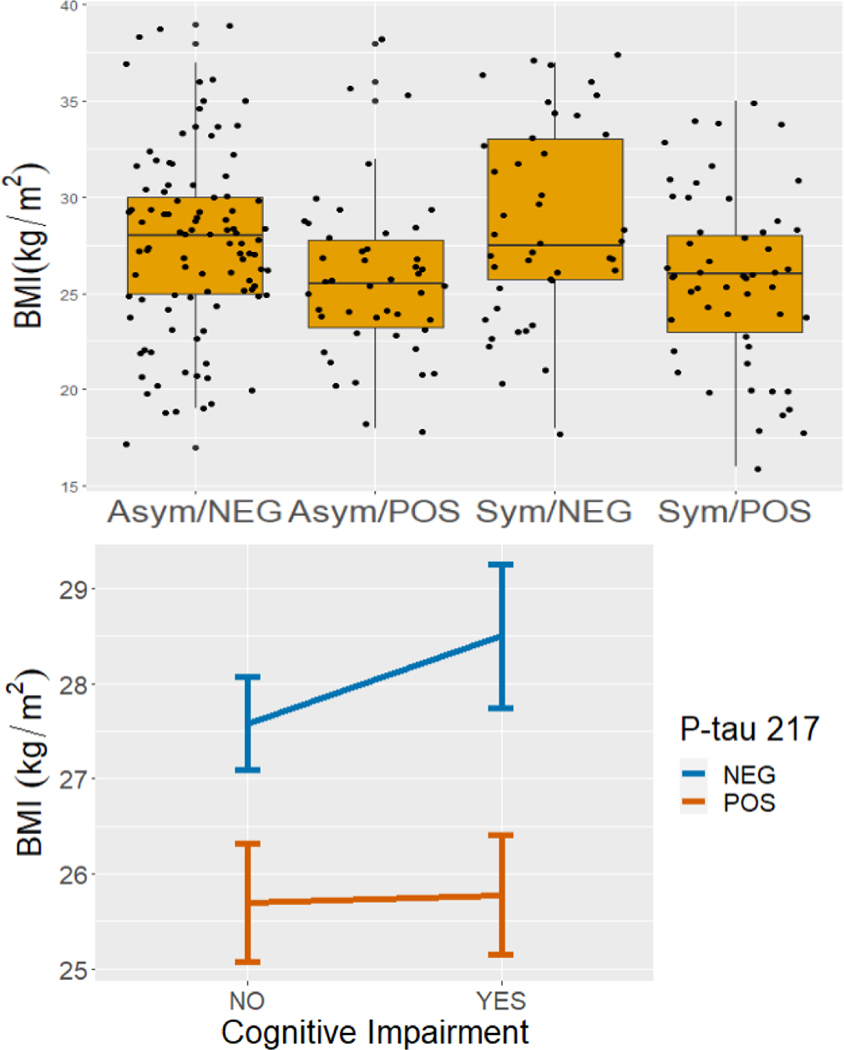
BMI by cognitive symptoms and P-tau 217 level. A. Boxplots show variation in BMI across the four groups defined by cognitive symptoms and P-tau 217 level. B. Interaction plots show mean BMI by cognitive impairment and P-tau 217 level. Error bars represent standard error of the mean. Asym = asymptomatic, Sym = symptomatic, POS = positive, NEG = negative.

**Figure 3.**
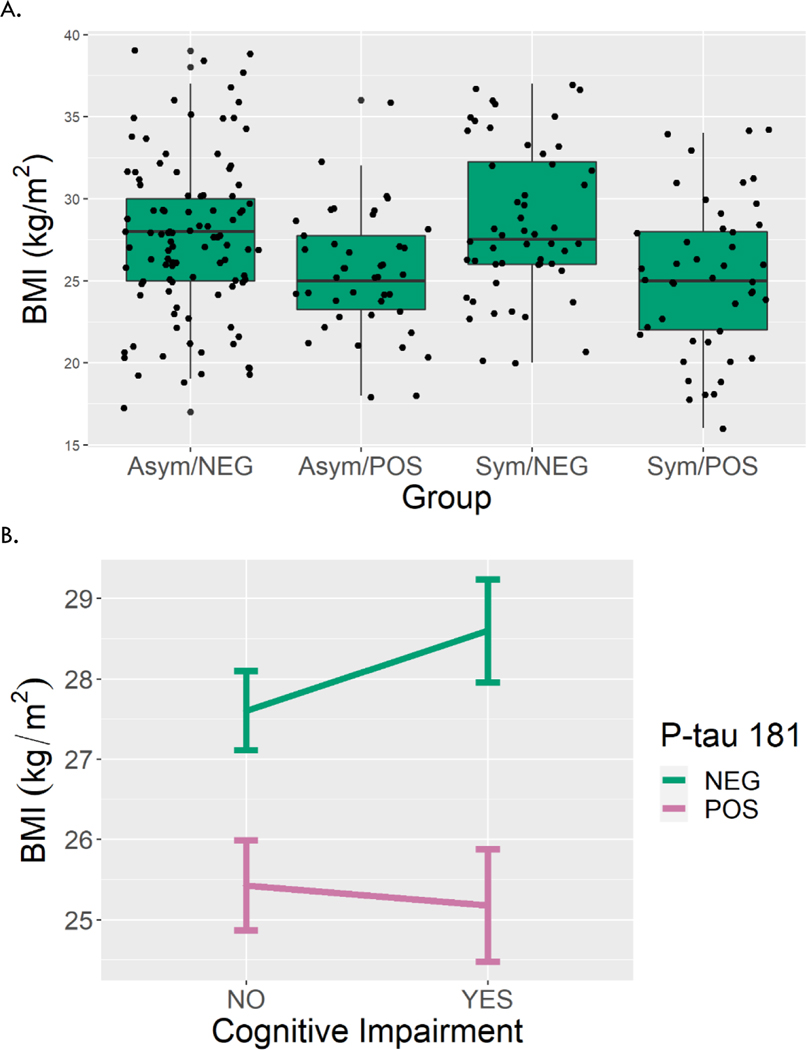
BMI by cognitive symptoms and P-tau 181 level. A. Boxplots show variation in BMI across the four groups defined by cognitive symptoms and P-tau 181 level. B. Interaction plots show mean BMI by cognitive impairment and P-tau 181 level. Error bars represent standard error of the mean. Asym = asymptomatic, Sym = symptomatic, POS = positive, NEG = negative.

Presence of cognitive symptoms did not influence the association of P-tau 217 (−0.84 kg/m^2^, p = 0.51) or P-tau 181 (−1.24 kg/m^2^, p = 0.33) with BMI. Symptomatic and asymptomatic participants had similar BMI (Figure 2; 0.92 kg/m^2^, p = 0.28 in the interaction model with P-tau 217; 0.99 kg/m^2^, p = 0.21 in model with P-tau 181). Individuals with elevated levels of P-tau 217 (−1.89 kg/m^2^, p = 0.025) and P-tau 181 (−2.18 kg/m^2^, p = 0.011) considered positive had lower BMI than individuals with lower P-tau levels (Figure 2, 3) considered negative. This association remained significant after adjustment for age, sex, ethnicity, education and *APOE-e4* status (−2.39 kg/m^2^, p = 0.005 for P-tau 217; −1.90 kg/m^2^, p = 0.028 for P-tau 181).

**Figure 4.**
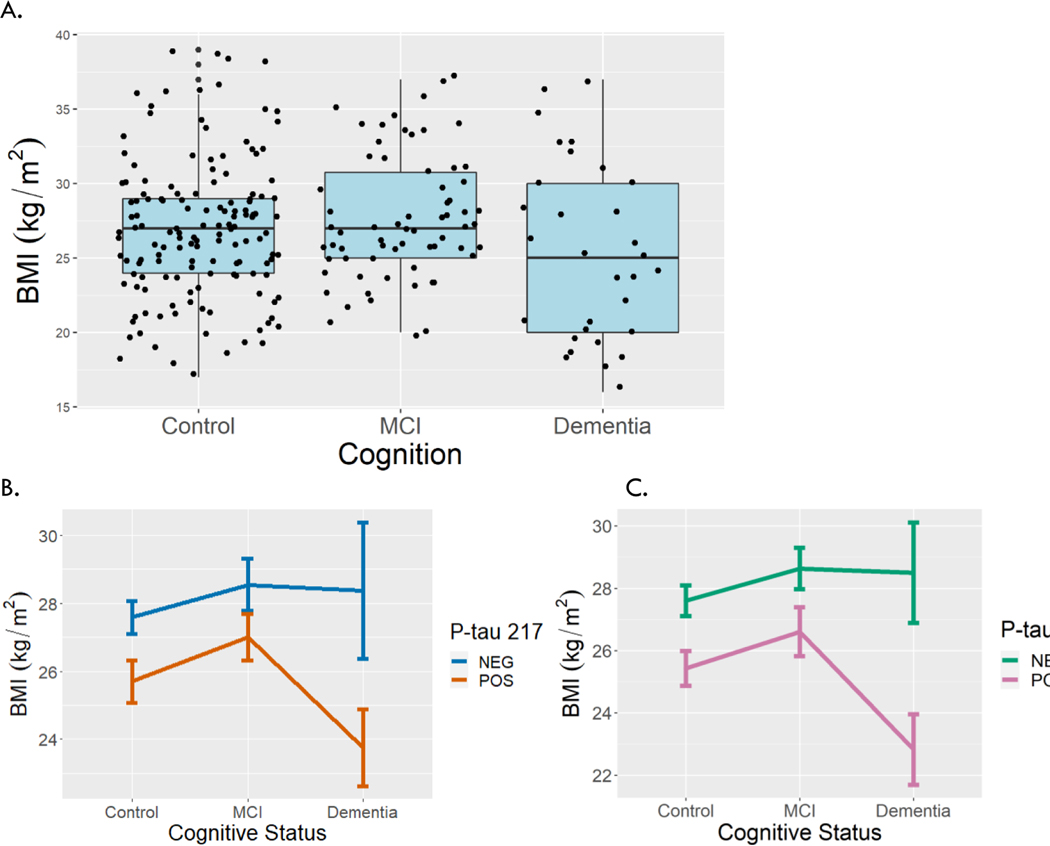
Variation in BMI by level of cognitive impairment and P-tau. Boxplots (A) show variation in BMI by level of cognitive impairment (control, MCI, dementia). Interaction plots show mean BMI by level of cognitive impairment and P-tau 217 (B) or P-tau 181 (C) level. Error bars represent standard error of the mean. MCI = mild cognitive impairment.

In an unadjusted model, BMI did not differ in participants with dementia (excluding MCI) (−1.59 kg/m^2^, p=0.09) or MCI (0.79 kg/m^2^, p=0.26) compared to controls (Figure 4). After adjusting for covariates as above, those with dementia had lower BMI than controls (−2.06 kg/m^2^, p=0.04). However, dementia was not associated with BMI after including P-tau 217 (−1.40 kg/m^2^, p=0.16) or 181 (−1.44 kg/m^2^, p=0.15) in the model.

**Figure 5.**
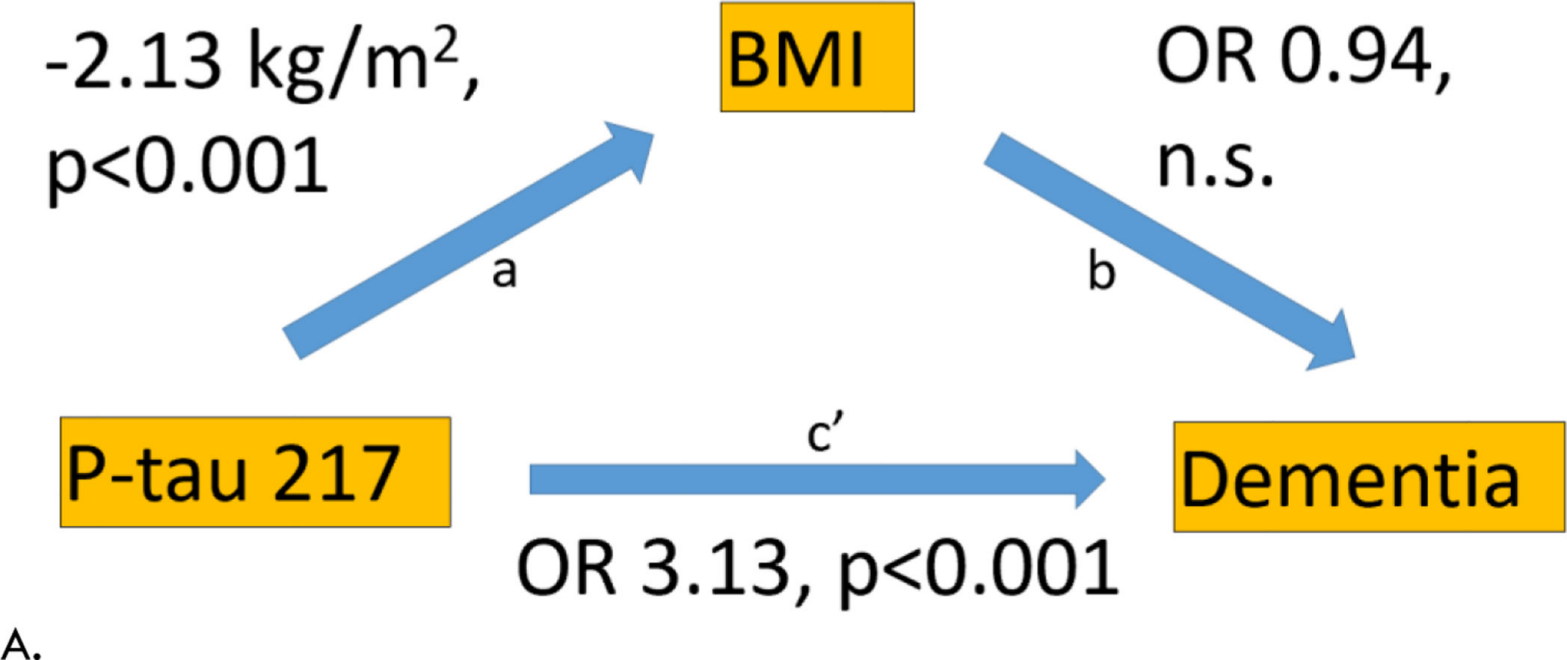

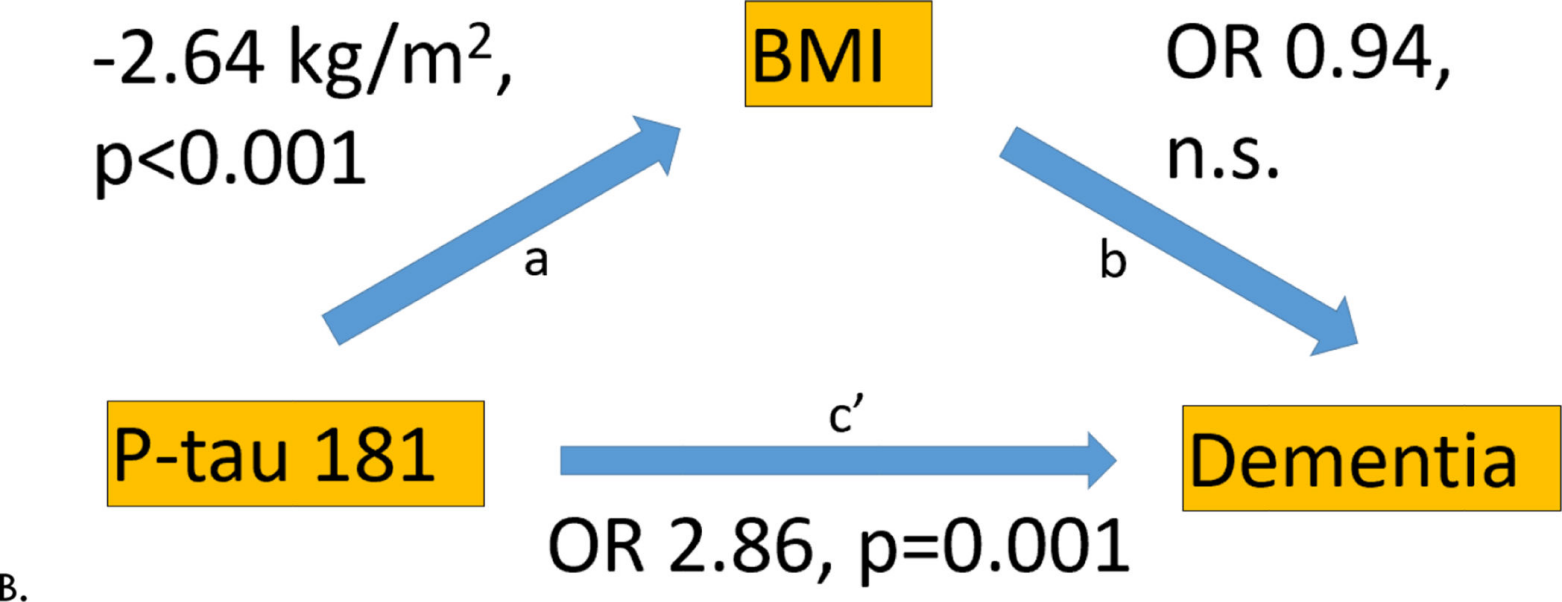
Mediation Analysis Analysis of BMI as a mediator of the effect of P-tau 217 (A) and P-tau 181 (B) on dementia (excluding MCI). a) Effect of P-tau level on BMI using linear regression. b) Effect of BMI on risk of dementia using logistic regression. c’) Effect of P-tau level on risk of dementia using logistic regression. OR = odds ratio, n.s. = not significant.

We also evaluated BMI as a mediator of the effect of P-tau 217 and 181 on risk of dementia using linear and logistic regressions. P-tau 217 and 181 positive participants had higher risk of dementia compared to negative participants (Figure 5). P-tau positive participants also had lower BMI compared to negative individuals (Figure 5). However, BMI did not influence risk of dementia after adjusting for P-tau 217 or 181 level (Figure 5). Therefore, BMI did not mediate the effect of P-tau 217 or 181 on dementia risk.

## Discussion

We investigated the relationship between antecedent conditions and risk factors and AD defined by blood-based biomarkers, P-tau 217 and P-tau 181, and comparing four groups: Asym/NEG, Asym/POS, Sym/NEG, Sym/POS as defined earlier. The goal was to determine which factors, if any, explained discordance between biomarker and clinically determined diagnoses. Here we found that P-tau (217 and 181) positive individuals had lower BMI than P-tau negative individuals, regardless of whether they had cognitive impairment. We also found that participants with AD in our group had lower BMI compared to controls after adjusting for several covariates. The frequency of vascular and other risk factors (diabetes, hypertension, heart disease, head injury) did not vary between the four groups.

To determine whether BMI affected the relationship between plasma P-tau (181 or 217) and AD, we used a mediation analysis. This analysis indicated that BMI was associated with plasma P-tau (181 or 217) levels and, independently BMI was also associated with dementia. Thus, BMI was unlikely to mediate the relationship between P-tau and dementia in our group. Nonetheless, if it is accepted that plasma P-tau 181 or 217 are part of the biological process defining AD then elevated plasma P-tau 181 or 217 levels even in asymptomatic individuals must be considered a component of AD pathogenesis as well as changes in body weight.

Previous studies have shown an increased risk of dementia associated with overweight in midlife and underweight in late life^20^. Studies have also found lower BMI in individuals with AD pathology^21^ and AD CSF profiles^22–25^. There are several possible hypotheses to explain these relationships. One is that BMI is a confounding factor^5,26^. We did not find support for this hypothesis in this study. In this group of individuals, BMI was related to P-tau but not dementia. When separating MCI from dementia and adjusting for covariates, those with dementia did have lower BMI compared to controls. However, this association disappeared after adjusting for P-tau.

It has been suggested that obesity without other signs of metabolic syndrome may be protective against dementia^22,23^. However, several biases undermine the evidence base of this hypothesis, including attrition, survivor, and reverse causality bias^27^. One explanation with growing acceptance is that weight loss is a consequence of AD^28,29^, rather than a risk factor. For example, one study showed higher BMI was associated with increased dementia risk when measured over 20 years before dementia diagnosis, but this association was reversed when it was measured less than 10 years before diagnosis, suggesting a reverse causality effect^8^.

A decline in BMI may occur before the onset of cognitive symptoms of AD. For example, a previous study of a larger group of individuals in the WHICAP cohort showed that participants lost 0.21 kg/m^2^ annually in the years leading up to diagnosis of dementia^30^. This was also suggested in the current study as indicated by the association of P-tau 181 and 217 even in asymptomatic individuals. Reduced BMI has also been found in those with AD biomarker CSF signatures regardless of the level of cognitive impairment^24,25^, including in unimpaired individuals^24^. In the current study, those with positive biomarkers had low BMI regardless of whether there was cognitive impairment, which was consistent with this observation. However, with our cross-sectional study, we were not able to determine whether weight loss or accumulation of biomarkers came first. Longitudinal studies are needed to clarify this relationship by showing the relationship between biomarkers, clinical status and change in weight over time.

We hypothesized that the Sym/NEG group would have a higher proportion of individuals with vascular risk factors than the Sym/POS group, but we did not find support for this hypothesis. It is possible that the small number of such individuals limited our ability to assess vascular risk factors. Dementia is attributable to AD in an estimated 50–80% of patients^31–33^.

Cerebrovascular pathology often accompanies typical neuropathology of AD^39,40^. Even in the presence of cerebrovascular pathology in AD, plasma P-tau181 and P-tau217 can discriminate AD from other age-related dementias and healthy controls^41^. The heterogeneity of pathology in clinically diagnosed AD reinforces the need to use blood-based biomarkers for research and clinical practice^42^.

The relationship of AD with vascular risk factors and cerebrovascular pathology is complex. In cross-sectional analysis, there is a consistent association between pathology of AD and of cerebrovascular disease^43–46^. The *APOE-e4* allele increases risk for both cerebrovascular disease and AD^47,48^. Several studies evaluating for associations between vascular comorbidities and AD molecular or imaging biomarkers were negative^11,49–52^. However, several large studies have shown associations of AD biomarkers and imaging markers of cerebrovascular disease, particularly white matter hyperintensity^53–55^. For example, in a study of 200 participants, plasma P-tau 181 and 217 were associated with increased white matter hyperintensity and lower white matter microstructural integrity^55^.

Our study is limited by the use of dichotomous, rather than continuous measures of cognitive status and biomarkers, which may be unable to show the full picture of the relationship between biomarkers, cognition, and vascular disease. In addition, analysis of categorical variables (such as peripheral vascular disease and head injury) was limited by sample size in comparison to continuous variables (such as BMI and Hb A1C). Our power was also limited by the low frequency of some conditions (like head injury) in our group. In addition, while we considered each independent variable separately, considering several together, as in a score, may give a clearer view of vascular risk. In addition, the relationship between biomarkers, clinical status and risk factors may change over time. Longitudinal studies, and those which include other biomarkers such as NfL and glial fibrillary acidic protein, may help clarify these relationships.

The main strength of our study is our community-based multi-ethnic cohort, which contributes to the generalizability of our analyses. The use of P-tau thresholds which correspond to pathologic data in the same population improves the internal validity of our results. However, it may limit their applicability to other study populations. Further research is necessary to develop additional biomarkers or combinations of biomarkers in plasma that may have still better sensitivity and specificity, and to examine relationships between biomarkers and comorbidities within ethnic groups.

Taken together this study revealed an inverse association of BMI with plasma P-tau concentrations, regardless of cognitive function, in a community-based cohort. Longitudinal studies, including multiethnic diverse populations, are necessary to clarify the relationship between blood-based biomarkers, BMI, and other comorbidities.

## Conclusions

The use of plasma-based biomarkers to support the clinical diagnosis of AD provides an avenue to establish high diagnostic accuracy at a relatively low cost and in a fashion acceptable to patients, families, and physicians. However, despite using rigorous criteria for the clinical diagnosis of AD, there exists patients with dementia who will be biomarker negative as well as asymptomatic individuals who are biomarker positive. One could argue that symptomatic patients have other forms of dementia and that asymptomatic patients are in preclinical stages of the disease. However, a sustained effort to determine the cause of this discordance is essential because plasma-based biomarkers will greatly advance the diagnosis and treatment of AD and other dementias in clinical practice and research.

## Figures and Tables

**Table 1.** Medical conditions across four groups determined by P-tau 217 and presence of cognitive impairment.

P-tau 217 group	Asym/NEG	Asym/POS	Sym/NEG	Sym/POS	Total	Statistic	P-value
**N**	119	54	60	67	300		
**Age**	81 (6.4)	81 (6.2)	82 (6.4)	84 (6.5)	82 (6.5)		
**Women**	81 (68%)	31 (57%)	42 (70%)	46 (69%)	200 (67%)		
**Ethnicity**							
non-Hispanic White	46 (39%)	18 (33%)	14 (23%)	22 (33%)	100 (33%)		
non-Hispanic Black	38 (32%)	17 (31%)	20 (33%)	25 (37%)	100 (33%)		
Hispanic	35 (29%)	19 (35%)	26 (43%)	20 (30%)	100 (33%)		
**BMI** ^a^	28 (4.8)	26 (4.2)	29 (5.0)	26 (4.6)	27 (4.8)	F-statistic = 4.4	0.0048*
**Diabetes Mellitus**	17 (15%)	10 (19%)	11 (23%)	10 (17%)	48 (17%)		
Hb A1C^b^	6 (0.7)	6 (0.7)	6 (0.7)	6 (0.6)	6 (0.7)	F-statistic = 1.2	0.29
**Hypertension**	102 (86%)	44 (81%)	53 (88%)	62 (93%)	261 (87%)	Chi square = 3.5	0.32
**Peripheral Vascular**							
**Disease** ^c^	45 (38%)	18 (33%)	27 (46%)	27 (41%)	117 (39%)	Chi square = 2.0	0.57
**Heart Disease**	53 (45%)	23 (43%)	28 (47%)	29 (43%)	133 (44%)	Chi square = 0.23	0.97
**Head Injury**	15 (13%)	5 (9%)	7 (12%)	7 (10%)	34 (11%)	Chi square = 0.48	0.92

Abbreviations: Asym = asymptomatic. Sym = symptomatic. POS = positive. NEG = negative. Categorical variables presented as n (%), and continuous variables presented as mean (SD).

* P-values significant after adjustment for multiple comparisons are shown in bold.

a BMI available for 240 participants.

b Hb A1C, excluding extreme outliers (>3 interquartile ranges above 3^rd^ quartile or below 1^st^ quartile), available for 274 participants.

c Peripheral vascular disease available for 297 participants.

* Significant after correction for multiple comparisons.

**Table 2.** Medical conditions across four groups determined by P-tau 181 and presence of cognitive impairment.

P-tau 181 group	Asym/NEG	Asym/POS	Sym/NEG	Sym/POS	Total	Statistic	P-value
**N**	123	49	68	59	299		
**Age**	80 (6.3)	82 (6.2)	82 (6.6)	84 (6.4)	82 (6.5)		
**Women**	87 (71%)	24 (49%)	45 (66%)	43 (73%)	199 (67%)		
**Ethnicity**							
non-Hispanic White	45 (37%)	19 (39%)	18 (26%)	18 (31%)	100 (33%)		
non-Hispanic Black	39 (32%)	15 (31%)	21 (31%)	24 (41%)	99 (33%)		
Hispanic	39 (32%)	15 (31%)	29 (43%)	17 (29%)	100 (33%)		
**BMI** ^a^	28 (5.0)	25 (3.6)	29 (4.6)	25 (4.7)	27 (4.8)	F statistic = 6.6	0.000277*
**Diabetes Mellitus**	19 (16%)	8 (16%)	12 (22%)	9 (17%)	48 (18%)		
Hb A1C^b^	6 (0.7)	6 (0.6)	6 (0.7)	6 (0.6)	6 (0.7)	F statistic = 0.32	0.81
**Hypertension**	106 (86%)	39 (80%)	60 (88%)	55 (93%)	260 (87%)	Chi square = 4.5	0.208
**Peripheral Vascular**							
**Disease** ^c^	45 (37%)	18 (37%)	31 (46%)	23 (40%)	117 (39%)	Chi square = 1.9	0.598
**Heart Disease**	53 (43%)	23 (47%)	32 (47%)	25 (42%)	133 (44%)	Chi square = 0.51	0.918
**Head Injury**	16 (13%)	4 (8%)	7 (10%)	7 (12%)	34 (11%)	Chi square = 0.92	0.821

Abbreviations: Asym = asymptomatic. Sym = symptomatic. POS = positive. NEG = negative. Categorical variables presented as n (%), and continuous variables presented as mean (SD).

* P-values significant after adjustment for multiple comparisons are shown in bold.

a BMI available for 241 participants.

b Hb A1C, excluding extreme outliers (>3 interquartile ranges above 3^rd^ quartile or below 1^st^ quartile), available for 275 participants.

c Peripheral vascular disease available for 298 participants.

* Significant after correction for multiple comparisons.
